# Assessing Calcified Coronary Lesions to Guide Percutaneous Coronary Intervention: From Angiography to Intracoronary Imaging

**DOI:** 10.3390/tomography12100148

**Published:** 2026-10-03

**Authors:** Sotirios C. Kotoulas, Andreas S. Triantafyllis, Athanasios C. Kotoulas, Konstantinos Antoniades, Ibraheem Aqueel, Agoritsa-Maria Pervana, Nikolaos Papaioannou, Katerina Peteinaki, Nikolaos Tsiamis, Nestoras Kontogiannis, Pavlos Tsinivizov, Leonidas E. Poulimenos

**Affiliations:** 1 Department of Cardiology, “Asklepeion” General Hospital of Voula, 16673 Athens, Greece; soter96@icloud.com (S.C.K.); thanassiskot@gmail.com (A.C.K.); konstantinos.a@hotmail.com (K.A.); ibraheemaqeel@yahoo.com (I.A.); riapervana@gmail.com (A.-M.P.); nickpapaiwannou@hotmail.com (N.P.); kpeteinaki@yahoo.com (K.P.); kontonest@gmail.com (N.K.); pavlost1980@gmail.com (P.T.); leonp@otenet.gr (L.E.P.); 2 Department of Cardiology, Sismanoglio General Hospital, 15126 Athens, Greece; nik.tsiamis@gmail.com

**Keywords:** coronary artery calcification, intracoronary imaging, intravascular ultrasound, optical coherence tomography, NIRS-IVUS, high-frequency optical coherence tomography, coronary computed tomography angiography, calcium scoring, percutaneous coronary intervention, stent underexpansion

## Abstract

**Simple Summary:**

Calcium in the coronary arteries makes them stiff and difficult to treat, and the amount, depth, and pattern of calcium strongly influence whether an intracoronary device can be delivered successfully. The severity of coronary calcium is a strong predictor of worse outcomes during percutaneous coronary intervention. Intracoronary calcium burden can now be evaluated with several imaging methods, ranging from standard angiography and computed tomography to intravascular devices that provide perspectives from inside the artery. Each method reveals different features of the calcium, such as how thick it is or whether it contains fatty material. This review explains how these imaging tools work, what each adds, and how combining them helps operators choose the safest and most effective way to prepare a calcified artery before intervention, with the aim of improving outcomes.

**Abstract:**

**Background/Objectives:** Coronary artery calcification is a leading cause of stent failure and a strong predictor of adverse outcomes after percutaneous coronary intervention (PCI). Accurate characterization of calcium burden, depth, and morphology is central to procedural planning, targeted treatment, and better outcomes. This review summarizes the contemporary armamentarium for imaging calcified coronary lesions, from coronary angiography and computed tomography to advanced intracoronary imaging, and how it facilitates treatment. **Methods:** We synthesized evidence from landmark studies, validated imaging scoring systems, registries, and current guideline and consensus documents addressing the assessment and imaging-guided treatment of coronary calcification. **Results:** Invasive angiography shows poor diagnostic accuracy for coronary calcium detection, characterization, and quantification. Coronary CT angiography noninvasively quantifies calcium (Agatston score and three-dimensional volume) and supports preprocedural planning, although it may overestimate calcium volume. Intravascular imaging by means of intravascular ultrasound (IVUS) and optical coherence tomography (OCT) effectively detects coronary calcium. Their use is associated with improved clinical outcomes compared with sole angiographic guidance. The addition of near-infrared spectroscopy (NIRS-IVUS) characterizes lipids within calcified plaque and high-risk calcified nodules. OCT provides the highest spatial resolution and uniquely measures calcium thickness and longitudinal calcium length, while the utilization of artificial intelligence further enhances calcium assessment. Integration of these modalities into a morphology-based, imaging-guided approach facilitates calcium detection and modification and supports PCI optimization. **Conclusions:** Intravascular imaging, along with coronary CT angiography, has transformed the assessment of calcified coronary lesions. Combining their strengths enables precise, lesion-specific preparation; randomized trials show that intravascular imaging guidance improves outcomes in complex PCI, while CT-guided PCI planning is emerging as a complementary strategy whose clinical value is now being tested in randomized trials.

## 1. Introduction

Coronary artery calcification is a hallmark of advanced atherosclerosis and a major determinant of technical difficulty and worse clinical outcomes of percutaneous coronary intervention (PCI). Population aging is sharply increasing the prevalence of severely calcified lesions during PCI. Calcified plaques are rigid and poorly compliant, leading to impaired lesion crossing, balloon expansion, stent delivery, and stent deployment; intravascular imaging shows that stent underexpansion and a smaller minimal stent area are associated with higher rates of stent thrombosis and in-stent restenosis [1,2,3], and pooled analyses have demonstrated that moderate to severe calcification is independently associated with higher rates of ischemic events and major adverse cardiac events following PCI [4,5].

The decision of whether and how to modify calcium depends not just on the presence of calcium but on its burden, circumferential arc, thickness, depth, and morphology. These parameters are captured imperfectly by angiography [6,7]. Over the past two decades, the assessment of calcified coronary lesions has evolved from a purely angiographic sign into a multimodality discipline spanning noninvasive computed tomography and high-resolution intracoronary imaging [8]. This review provides a comprehensive overview of contemporary imaging modalities, from coronary angiography to coronary CT angiography (CCTA), intravascular ultrasound (IVUS), near-infrared spectroscopy–IVUS (NIRS-IVUS), and optical coherence tomography (OCT), including high-frequency OCT (HF-OCT), and describes how their complementary strengths can be orchestrated to guide calcium modification, optimize PCI and improve outcomes.

## 2. Materials and Methods

This narrative review synthesizes the current evidence on multimodality imaging for the assessment of coronary artery calcification and its role in guiding percutaneous coronary intervention (PCI). We searched PubMed/MEDLINE, Scopus, and the Cochrane Library for English-language articles published from database inception through July 2026, using combinations of the following search terms: “coronary artery calcification”, “coronary calcification”, “intravascular imaging”, “intracoronary imaging”, “intravascular ultrasound”, “near-infrared spectroscopy”, “optical coherence tomography”, “high-frequency optical coherence tomography”, “coronary CT angiography”, “calcium score”, “stent underexpansion”, and “calcium modification”. Priority was given to landmark imaging studies, pivotal clinical trials, high-quality observational studies, large registries, and contemporary guideline and consensus documents. The reference lists of relevant publications were also screened to identify additional eligible studies. As this was a narrative rather than a systematic review, studies were selected on the basis of their clinical relevance, methodological quality, and contribution to the topic. No formal risk-of-bias assessment was performed.

## 3. Pathophysiology and Morphology of Coronary Calcification

Coronary calcification is an actively regulated process involving osteoblast-like transformation of vascular smooth muscle cells under the influence of oxidative stress, inflammation, and disordered mineral metabolism [9,10,11,12,13]. These mechanisms promote the deposition of hydroxyapatite crystals within the arterial wall, contributing to plaque progression and alterations in vascular biomechanics.

Two morphological patterns are recognized. Microcalcifications, typically measuring <15 µm, although larger aggregates up to 50 μm have been described, develop within the lipid-rich and necrotic regions of atherosclerotic plaques and within the fibrous cap. When located within a thin fibrous cap, particularly when closely clustered, they can act as local stress concentrators and increase circumferential stress on the cap, thereby promoting plaque instability and rupture [14,15,16]. Individual microcalcifications lie at or below the axial resolution of OCT (10–20 μm) and below its lateral resolution (20–40 μm) and are well below the resolution of conventional IVUS (approximately 100–150 μm axially). Consequently, individual histological microcalcifications cannot be reliably resolved by current intracoronary imaging. OCT may identify small calcific foci or aggregates, but these do not necessarily correspond to individual microcalcifications. Very small calcific deposits may be difficult to distinguish from other punctate plaque features. Histology and high-resolution micro-computed tomography therefore remain the reference methods for their morphological assessment [16].

Microcalcification is associated with biologically active, inflammatory plaque and with morphological features of plaque vulnerability, including plaque rupture and acute coronary syndromes. Active mineralization can be visualized noninvasively with 18F-sodium fluoride positron emission tomography (18F-NaF PET), which preferentially accumulates at exposed hydroxyapatite crystal surfaces in areas of active calcification. Coronary 18F-NaF uptake localizes to culprit lesions and to plaques exhibiting high-risk features [17], and independently predicts subsequent myocardial infarction in patients with established coronary artery disease [18]. Microcalcification primarily reflects active plaque biology and vulnerability rather than the mechanical resistance associated with extensive macrocalcification. Its potential clinical value lies in assessment of plaque activity and cardiovascular risk rather than in guiding mechanical lesion preparation for PCI, although the role of microcalcification imaging in directing preventive therapy remains investigational. In contrast, macrocalcifications form dense larger sheets or nodules and are commonly associated with stable atherosclerotic plaques but present substantial technical challenges during PCI due to reduced vessel compliance [19].

From an interventional perspective, the distinction between intimal (superficial) and medial (deep) calcification is particularly important. Intimal calcification develops within atherosclerotic plaques and represents the principal target of coronary intervention, whereas medial calcification (Mönckeberg sclerosis) primarily affects the medial layer of the vessel wall, contributes to arterial stiffening, and is encountered more frequently in patients with diabetes mellitus and chronic kidney disease [20]. Although both patterns may coexist, differentiating them has important procedural implications because superficial calcium is generally more amenable to atherectomy-based plaque modification, whereas deeper medial calcium is more effectively fractured using intravascular lithotripsy [21].

A further high-risk morphological entity is the calcified nodule, characterized by eruptive nodular calcium protruding into the coronary lumen following disruption of the fibrous cap [22,23,24]. Calcified nodules are associated with acute coronary syndromes, impaired device delivery, incomplete stent expansion, and increased procedural complexity. Their recognition is therefore important both for procedural planning and for selecting the most appropriate calcium-modification strategy.

Importantly, the procedural behavior of calcified coronary lesions depends not only on the overall calcium burden but also on its three-dimensional morphology, including calcium thickness, circumferential arc, longitudinal extent, depth, and distribution. These characteristics determine lesion compliance, the likelihood of adequate balloon expansion, the probability of calcium fracture during lesion preparation, and ultimately the risk of stent underexpansion. Consequently, contemporary coronary imaging has evolved beyond simple calcium detection toward comprehensive morphological characterization that directly informs procedural planning and device selection [25].

Because these morphological features cannot be adequately characterized by conventional angiography alone, contemporary assessment increasingly relies on complementary noninvasive and intracoronary imaging modalities. The ability to quantify calcium arc, thickness, depth, longitudinal extent, and plaque composition has become central to selecting the optimal calcium-modification strategy and achieving durable PCI results.

## 4. Impact of Coronary Calcification on Percutaneous Coronary Intervention

Coronary calcification is not merely a marker of disease burden; it actively impairs every stage of PCI. At the outset, heavily calcified and tortuous segments hinder the advancement of guidewires and may limit the crossing of balloons, intravascular imaging catheters, atherectomy devices and stents [26,27]. Even after successful wire crossing, conventional balloons may fail to dilate the lesion, the so-called balloon-undilatable lesion, encountered in approximately 5 to 10% of calcified procedures [27,28]. When dilation is achieved, rigid calcium causes asymmetric, incomplete stent expansion, with stent expansion reduced as much as 60 to 70% relative to adjacent noncalcified segments [20,29]. Incomplete lesion expansion may result in inadequate stent apposition, further increasing the risk of adverse procedural and long-term outcomes. Calcified lesions also carry a higher incidence of procedural complications, including coronary dissection, perforation, device entrapment and slow flow or no reflow from distal embolization of calcific debris [30,31].

These acute mechanical consequences translate into worse long-term outcomes. Stent underexpansion is the strongest predictor of stent thrombosis, a complication with mortality exceeding 40% in some series [32,33], while stent malapposition and geographic miss promote in-stent restenosis and neoatherosclerosis [33,34].

The adverse prognostic impact of calcification has been quantified across large registries and pooled analyses. A patient-level pooled analysis of seven contemporary stent trials, comprising more than 20,000 patients, found that moderate to severe calcification independently increased all-cause mortality by approximately 50% and major adverse cardiac events by approximately 40% [4], and a pooled analysis of the HORIZONS AMI and ACUITY trials linked calcification to excess ischemic events after PCI in acute coronary syndromes [5]. In a prospective registry of more than 10,000 patients, moderate to severe calcification independently predicted target vessel revascularization and composite events at two years [35], and even with second-generation drug-eluting stents, patients with significant calcification experienced substantially higher five-year rates of target lesion failure [36,37]; a single-center study similarly reported markedly higher one-year event rates in severely calcified lesions [38]. In the multicenter COBIS II registry of 2897 patients, moderate or severe calcification of bifurcation lesions increased the adjusted risk of target lesion failure (hazard ratio [HR] 1.31), driven mainly by repeat revascularization [39].

Despite substantial advances in calcium-modification technologies, patients with severe coronary calcification continue to represent one of the highest risk populations undergoing PCI. Contemporary registries of patients treated with dedicated calcium modification confirm that event rates remain high in this population: in a series of 632 patients undergoing rotational atherectomy for predominantly severe calcification, three-year mortality reached 21.7% and target lesion revascularization 13.0%, reflecting the high-risk lesion and patient profile rather than a failure of treatment [40]. This continuum, from impaired lesion crossing and preparation to stent underexpansion, procedural complications, and adverse long-term outcomes, explains why accurate assessment and modification of calcium before stenting is crucial during contemporary PCI [41].

## 5. Coronary Angiography: The Starting Point

Coronary angiography remains the most widely used and immediately available method for assessing calcification, as it provides real-time visualization of coronary anatomy, lesion severity, coronary flow and device positioning. It allows an initial assessment of lesion complexity and procedural planning.

Unfortunately, angiography has limited sensitivity for detecting and characterizing coronary calcifications. In the classic intravascular ultrasound study by Mintz et al. of 1155 lesions, angiography identified calcium in only 38% of lesions, whereas IVUS detected calcium in 73%. Angiographic sensitivity increased with the circumferential extent of calcium, reaching 60% for three-quadrant and 85% for four-quadrant calcification [1]. Two decades later, Wang et al. compared the three modalities in vivo in 440 lesions undergoing OCT- and IVUS-guided stent implantation. Because no histological reference standard is available in vivo, intravascular imaging served as the comparator for angiography. Calcium was detected in 40.2% of lesions by angiography, 82.7% by IVUS and 76.8% by OCT, and in 21.6% of lesions with an IVUS calcium arc > 180°, angiography detected no calcium at all. Notably, OCT either failed to visualize calcium or underestimated its maximum arc by >90° in 13.2% of lesions with IVUS-detected calcium, predominantly because of superficial signal attenuation [8]. Histologically validated accuracy is available only from ex vivo studies. In a validation study, OCT identified fibrocalcific plaque with a sensitivity of 95–96% and a specificity of 97% [42]. IVUS demonstrates high sensitivity and specificity for macroscopic calcification, although its accuracy depends on the size and the histological pattern of calcium. Furthermore, acoustic shadowing behind calcium prevents reliable assessment of calcium thickness and obscures tissue beyond its leading edge [25,43].

These limitations are inherent to angiography as a two-dimensional luminographic technique. It detects only sufficiently dense radiopaque calcium and cannot reliably determine calcium thickness, circumferential arc, depth, or plaque composition. Consequently, lesions that appear only moderately calcified angiographically may harbor severe circumferential calcium on intravascular imaging and therefore be at increased risk of inadequate lesion preparation and stent underexpansion.

Severe angiographic calcification, defined as radiopacities visible on both vessel walls without cardiac motion, should therefore be regarded as an indication for further assessment with intracoronary imaging rather than as a complete characterization of calcium severity as recommended by contemporary consensus [44].

Coronary angiography provides the starting point for evaluating calcified coronary lesions but should be complemented by advanced imaging modalities when detailed morphological assessment is required.

## 6. Computed Tomography: Noninvasive Assessment of Coronary Calcium

### 6.1. Coronary Artery Calcium Score

Non-contrast, electrocardiogram-gated cardiac computed tomography quantifies calcium using the Agatston scoring system, which weights each calcified voxel above 130 Hounsfield units (HU) according to its peak density. The resulting coronary artery calcium (CAC) score is a well-established marker of total coronary calcium burden and one of the most extensively validated tools for cardiovascular risk stratification in asymptomatic individuals. A CAC score of zero is associated with a very low 10-year risk of cardiovascular events, whereas scores above 400 indicate a high atherosclerotic burden and are strongly associated with obstructive coronary artery disease and adverse clinical outcomes [45,46,47,48].

Although the CAC score quantifies the overall burden of coronary calcification, it provides limited information regarding individual lesion characteristics, and its prognostic meaning is not determined by burden alone. The Agatston score is calculated as the product of the calcified plaque area and a density weighting factor (1, 2, 3 or 4 for a peak attenuation of 130–199, 200–299, 300–399 and ≥400 HU, respectively), summed across all lesions. Denser plaque is therefore up-weighted, and a higher score reflects both more extensive and denser calcification.

When calcium volume and density were analyzed separately in the Multi-Ethnic Study of Atherosclerosis (MESA), they behaved in opposite directions: calcium volume was strongly and positively associated with coronary and cardiovascular events (HR ≈ 1.8 per standard deviation [SD]), whereas, for any given volume, higher calcium density was independently associated with a lower risk of events (HR ≈ 0.7 per SD) [49,50]. This is not in conflict with the well-established association between a high Agatston score and obstructive disease and adverse outcomes. The Agatston score is dominated by plaque area and volume, which increase with total atherosclerotic burden and drive its prognostic value; the density term merely modulates it. Dense, sheet-like calcium represents a healed, quiescent stage of plaque evolution, whereas low-density calcium (spotty calcification and microcalcification) marks active inflammation and instability. The practical implications are twofold. For risk prediction, the Agatston score, by rewarding density, slightly underestimates the risk carried by extensive low-density calcification and overestimates that of dense mature calcium, which is why separate reporting of volume and density has been proposed [50]. For the interventionalist, the same dense calcium that is prognostically benign is precisely the calcium that resists balloon dilatation and requires modification before stenting.

The CAC score should be regarded as a marker of global atherosclerotic burden rather than a tool for procedural planning. It does not identify the lesion-specific distribution, circumferential arc, thickness or depth of calcium, nor can it determine the morphological characteristics that influence PCI strategy. These features require CCTA or intravascular imaging for accurate assessment [25].

### 6.2. Plaque and Lesion Characterization on Coronary CT Angiography

CCTA extends beyond calcium quantification to provide three-dimensional characterization of individual coronary plaques, allowing classification as calcified, non-calcified, or mixed and depicting the distribution and pattern of calcification, from discrete spotty deposits to extensive sheet-like calcium. Unlike non-contrast CAC scoring, CCTA simultaneously evaluates both calcified and non-calcified plaque components, providing a more comprehensive assessment of plaque morphology and overall coronary atherosclerotic burden.

Several adverse plaque characteristics can be identified on CCTA and have been consistently associated with an increased risk of future cardiovascular events, including low-attenuation plaque, positive remodelling, the napkin-ring sign, and spotty calcification [51]. The coexistence of multiple high-risk plaque features identifies lesions with greater biological activity and a higher likelihood of subsequent clinical events. In contrast, extensive confluent calcification generally reflects more advanced and stable atherosclerotic disease, although it frequently presents greater technical challenges during PCI.

CCTA provides information that extends beyond cardiovascular risk prediction by identifying lesion morphology before the procedure. Plaque composition, calcium distribution, vessel course, lesion length, and the relationship of calcification to adjacent non-calcified plaque can all be assessed noninvasively, facilitating procedural planning before invasive coronary angiography.

These findings have direct implications for revascularization. Lesion-level calcium burden on CCTA predicts both the need for calcium modification and the risk of stent underexpansion. Sekimoto et al. showed that a regional (lesion-specific) calcium score ≥ 453 on multidetector CT predicted the need for rotational atherectomy with 93% sensitivity and 88% specificity, and CT calcium arc ≥ 270° predicted the need for rotational atherectomy with 97% specificity [52]. More recently, a lesion-specific Agatston-type score of >200 on CCTA identified lesions with a 13-fold higher risk of stent underexpansion [53]. CCTA also defines the length of the calcified segment, its relationship to side branches and the presence of a calcified nodule, informing the choice between atherectomy, intravascular lithotripsy and balloon-based strategies, the selection of stent length and landing zones, and the recognition of diffusely calcified vessels with poor distal targets that may be better served by surgical revascularization. Conversely, low-attenuation plaque and the napkin-ring sign identify lesions prone to periprocedural myocardial infarction and distal embolization, prompting consideration of adjunctive measures. In this way, CCTA plaque characterization converts a diagnostic finding into a procedural plan: the likely need for a calcium-modification device, an imaging catheter and appropriate stent inventory can be anticipated before the patient reaches the catheterization laboratory rather than being determined ad hoc.

Recent advances in artificial intelligence (AI) and deep-learning algorithms have further expanded the capabilities of CCTA. Automated plaque analysis now enables rapid quantification of total, calcified, and non-calcified plaque volumes with good agreement compared with intravascular imaging while providing incremental prognostic information beyond conventional visual assessment [54,55].

### 6.3. Calcium Quantification and Preprocedural Planning

The principal advantage of CCTA lies in procedural planning before invasive coronary angiography. By providing three-dimensional visualization of calcium distribution throughout the coronary tree, CCTA allows early identification of heavily calcified lesions and facilitates planning of the interventional strategy. It also enables assessment of vessel size, lesion length, coronary tortuosity, and the anatomical distribution of calcification, all of which may influence vascular access, guide catheter selection, device choice, and the anticipated need for calcium-modification techniques.

In a direct comparison with OCT, CCTA-derived calcium volume overestimated the reference standard by approximately 60%, while maintaining good agreement for minimal lumen area [56]. Accordingly, CCTA should be regarded as a reliable tool for identifying extensive coronary calcification rather than for precise quantification of calcium volume.

The principal limitation of CCTA in heavily calcified vessels remains calcium blooming artifact, which exaggerates calcium dimensions and obscures the residual coronary lumen, potentially leading to overestimation of stenosis severity. Spatial resolution is the fundamental constraint: contemporary energy-integrating detector CT has an effective spatial resolution of approximately 0.4–0.6 mm (isotropic voxels of ~0.5 mm), an order of magnitude coarser than IVUS and almost two orders of magnitude coarser than OCT, so that calcium deposits of a few hundred micrometers are rendered as blooming that inflates their apparent size. Photon-counting detector CT (PCCT) in ultra-high-resolution mode achieves an in-plane spatial resolution of approximately 0.11 mm and substantially improved through-plane resolution [57], and, together with ultra-high-resolution image reconstruction, has substantially reduced blooming artifact and improved lumen visualization, resulting in more accurate assessment of calcified coronary stenoses [58].

Emerging technologies may further enhance the role of CCTA in procedural planning. CT-derived fractional flow reserve (CT-FFR) provides complementary functional assessment of lesion significance, while AI-assisted image analysis enables automated plaque quantification and risk stratification. Although these techniques continue to evolve, they have the potential to further refine patient selection and procedural planning for complex calcified coronary disease [59,60].

### 6.4. CT-Guided PCI: Current Status and near Future

The concept of CT-guided PCI extends CCTA from a diagnostic gatekeeper to an integral component of the procedure itself. In the current paradigm, CCTA is used before invasive coronary angiography to (1) select patients and lesions for PCI, in combination with CT-FFR; (2) plan the procedure, including vascular access, guide catheter and wire strategy, the anticipated need for calcium modification, stent length and diameter and the landing zones; and (3) co-register the CT data set with fluoroscopy in the catheterization laboratory to guide device positioning [61,62]. The P3 (Precise Percutaneous Coronary Intervention Plan) study validated CCTA-based virtual PCI planning against OCT, demonstrating excellent agreement for stent sizing and distal landing zone dimensions [61]. Separately, CCTA-based procedural planning has demonstrated clinical benefit in chronic total occlusion (CTO) PCI: in a trial of 400 patients, preprocedural CCTA guidance increased the rate of successful recanalization from 84.0% to 93.5% (*p* = 0.003) with fewer complications, largely through better characterization of proximal cap ambiguity, occlusion length, tortuosity and calcification [63]. For calcified lesions specifically, CCTA-derived lesion calcium scores and calcium arc predict the need for atherectomy and the risk of stent underexpansion [52,53], allowing calcium-modification devices and imaging catheters to be planned and made available in advance. A 2025 SCAI/SCCT expert opinion document now describes a structured workflow for CCTA-based PCI planning and identifies heavily calcified disease as one of its principal indications [62]. Two caveats apply. First, CCTA overestimates calcium volume, and its spatial resolution is inferior to intravascular imaging, so CT-based plans require confirmation by IVUS or OCT once the lesion has been prepared. Second, outside CTO PCI, the evidence that CT-guided planning improves clinical outcomes is currently limited. The ongoing Precise Procedural and PCI Plan (P4) trial (NCT05253677), which randomizes approximately 1090 patients to CCTA-guided procedural planning versus IVUS-guided PCI, will determine whether CT-based planning can improve PCI outcomes [64]. Combined with photon-counting CT and AI-based plaque quantification, CT-guided PCI has the potential to move much of the calcium assessment described in this review from the catheterization laboratory to the preprocedural planning stage, with intravascular imaging then used to confirm lesion preparation and optimize the final stent result.

## 7. Intravascular Ultrasound and NIRS-IVUS

### 7.1. Intravascular Ultrasound

IVUS uses high-frequency ultrasound (typically 20–60 MHz) to generate cross-sectional tomographic images of the coronary artery, enabling assessment of both the vessel lumen and the arterial wall. Spatial resolution depends on transducer frequency: at 40 MHz, the axial resolution is approximately 100–150 μm and the lateral resolution is 150–300 μm, improving to 40–60 μm axially with 60-MHz high-definition IVUS, while a penetration depth of 5–10 mm allows imaging of the full vessel wall [65]. Its greater tissue penetration compared with OCT allows visualization of the entire vessel architecture, making IVUS particularly useful in large vessels, ostial lesions, and diffuse coronary disease.

Calcium has a characteristic appearance on IVUS. Because it strongly reflects ultrasound waves, it is visualized as a bright hyperechoic arc accompanied by posterior acoustic shadowing, frequently associated with reverberation artifacts. Although this appearance allows calcium to be identified with high confidence, the acoustic shadow prevents visualization of tissue behind the calcified plaque, limiting direct measurement of calcium thickness [25,43].

Consequently, IVUS accurately assesses calcium arc, longitudinal length, and overall calcium distribution, but does not directly measure calcium thickness or consistently distinguish superficial from deep calcium beyond its leading edge [43]. These measurements are clinically important because increasing calcium arc and longitudinal extent are associated with a greater likelihood of incomplete stent expansion. In particular, a calcium arc > 270° has consistently been associated with impaired stent expansion following PCI [25,29].

Building on these observations, Zhang et al. developed and validated an IVUS-derived calcium score incorporating four independent predictors of stent underexpansion: (1) a superficial calcium arc > 270° extending over >5 mm, (2) circumferential (360°) calcium, (3) the presence of a calcified nodule, and (4) a reference vessel diameter < 3.5 mm. A score ≥ 2 identifies lesions at high risk of stent underexpansion, with excellent negative predictive value, and may assist in selecting lesions that require calcium-modification techniques before stent implantation [7].

Beyond calcium assessment, IVUS offers important practical advantages during PCI. Because ultrasound penetrates blood and does not require contrast-mediated blood clearance, image quality is maintained in large vessels, ostial lesions, left main disease, and hemodynamically unstable patients. IVUS also permits accurate measurement of vessel dimensions, plaque burden, and stent expansion throughout the procedure. Multiple randomized trials and meta-analyses have demonstrated that IVUS-guided PCI reduces major adverse cardiovascular events, target lesion revascularization, and stent thrombosis compared with angiography-guided PCI, supporting its routine use in complex coronary intervention [66,67].

### 7.2. Near-Infrared Spectroscopy–IVUS

NIRS-IVUS integrates structural and compositional plaque assessment within a single imaging catheter. While IVUS defines vessel architecture and calcium morphology, NIRS identifies lipid-rich plaque by measuring the maximum lipid core burden index over 4 mm, thereby complementing morphological assessment with information on plaque composition [68]. NIRS is a spectroscopic rather than a structural imaging technique: it does not independently provide structural measurements, samples only the innermost approximately 1 mm of the vessel wall and displays its output as a chemogram mapped by longitudinal and circumferential position onto the co-registered IVUS pullback, with the IVUS component providing the structural measurements.

This combined approach is particularly valuable in calcified coronary disease, where calcium and lipid frequently coexist within the same lesion. NIRS-IVUS identifies lipid-rich components within or adjacent to calcified plaques (“lipidic calcification”), a feature that cannot be detected by grayscale IVUS alone. Recognition of these lipid-rich calcified lesions may be clinically important, as they appear more susceptible to distal embolization and microvascular obstruction during PCI [68].

The lipid core burden index (LCBI) is the fraction of valid chemogram pixels with a high probability of lipid core, multiplied by 1000 (scale 0–1000); maxLCBI4mm is the highest LCBI found in any 4-mm segment, i.e., the lipid content of the most lipid-rich 4-mm segment. A maxLCBI4mm ≥ 400, corresponding to more than 40% of valid chemogram pixels being classified as lipid, was identified in the prospective Lipid-Rich Plaque study as the threshold above which both patients and individual non-culprit plaques carried a significantly increased risk of subsequent major adverse cardiac events [69], and this cut-off has since been adopted as the operational definition of a large lipid core. NIRS-IVUS also refines the evaluation of calcified nodules, an established high-risk plaque phenotype. Among patients presenting with acute coronary syndromes attributable to calcified nodules, a maxLCBI4mm ≥ 400 has been associated with a higher incidence of the no-reflow phenomenon and worse long-term clinical outcomes [70].

Although NIRS does not improve calcium quantification itself, it provides incremental information that complements IVUS-derived morphological assessment. By distinguishing predominantly calcified plaques from those containing a substantial lipid core, NIRS-IVUS enhances lesion characterization and may help identify lesions at increased risk of distal embolization during PCI.

## 8. Optical Coherence Tomography and High-Frequency OCT

### 8.1. Optical Coherence Tomography

OCT uses near-infrared light rather than ultrasound to generate high-resolution cross-sectional images of the coronary artery. With an axial resolution of approximately 10–20 µm, OCT provides the highest spatial resolution among currently available coronary imaging modalities, nearly ten times greater than IVUS. Unlike ultrasound, near-infrared light penetrates calcified tissue sufficiently to delineate its borders, allowing OCT to characterize calcium morphology with greater precision. On OCT, calcium appears as a signal-poor or heterogeneous region with sharply defined borders, enabling direct measurement of calcium thickness, in addition to calcium arc and longitudinal extent [71,72].

Against histology, OCT identifies calcified plaque with a sensitivity of 96% and a specificity of 97% [42]. In the in vivo comparison by Wang et al., however, OCT detected calcium in 76.8% of lesions versus 82.7% with IVUS, missing or underestimating calcium in 13.2% of IVUS-positive lesions because of superficial attenuation [8]; the strength of OCT therefore lies not in detection per se but in its resolution, which has enabled the development of OCT-derived calcium scoring systems that incorporate the morphological features most strongly associated with stent underexpansion. The Fujino OCT calcium score assigns points according to maximum calcium angle > 180° (2 points), calcium thickness > 0.5 mm (1 point), and calcium length > 5 mm (1 point). A maximum score of 4 identifies lesions at increased risk of stent underexpansion, with lower stent expansion compared with lesions with lower scores (78% versus 96% expansion) [6]. A revised OCT calcium score has subsequently been developed for severely calcified lesions in the contemporary drug-eluting stent (DES) era: it assigns one point each for a calcium arc > 270° extending over >3 mm, 360° calcium and a minimum calcium thickness > 0.30 mm (range 0–3), and stent expansion decreases stepwise with each additional point, with a score ≥ 2 showing a significantly greater risk of underexpansion and a score of 3 identifying the highest risk [73].

Beyond calcium quantification, OCT provides important procedural information during complex PCI. It is considered the reference intracoronary imaging modality for detailed characterization of calcified nodules [23] and enables direct assessment of calcium fracture following lesion modification, including the circumferential calcium fractures observed after intravascular lithotripsy [20]. OCT also facilitates stent optimization by allowing precise evaluation of stent expansion, apposition, and edge complications, with a low stent expansion index independently associated with adverse clinical outcomes [74].

The main limitations of OCT are related to its optical nature. Light penetration is limited in very thick or lipid-rich calcified plaques, where the deep calcium border may not be fully visualized. In addition, OCT requires transient displacement of blood using contrast injection, which may limit its use in patients with advanced renal dysfunction, hemodynamic instability, or situations where increased contrast volume administration is undesirable.

### 8.2. High-Frequency OCT and Emerging Software Technologies

HF-OCT is a recent technological advancement designed to improve the practicality and applicability of intracoronary OCT imaging. The Gentuity HF-OCT system (Gentuity LLC, Sudbury, MA, USA) uses a 1.8 F (0.6 mm) rapid-exchange micro-imaging catheter, approximately half the profile of conventional OCT catheters, with an axial resolution of ≤20 µm in tissue, a frame rate of 250 frames per second and a pullback speed of 100 mm/s, so that a 100-mm segment is imaged in one second; its extended scan range accommodates vessels from 1.3 to 6.0 mm in diameter [75]. These features facilitate image acquisition in complex coronary anatomy, including tortuous, heavily calcified and distal vessels, while preserving the high spatial resolution that distinguishes OCT.

Early clinical experience has been encouraging. In an initial feasibility study, HF-OCT demonstrated excellent deliverability, successful lesion crossability, and high-quality image acquisition before and after PCI across a broad range of vessel sizes, although severe vessel tortuosity reduced the length of analyzable images [75]. Subsequent studies have confirmed the feasibility of HF-OCT in large coronary arteries and other complex coronary anatomies, supporting its potential to expand the use of OCT-guided PCI [76]. However, larger prospective studies are still required to determine whether these technical advantages translate into improved procedural or clinical outcomes.

Advances in software have accompanied developments in imaging hardware. AI-assisted OCT analysis, exemplified by the Ultreon™ software platform (Abbott, North Chicago, IL, USA), automates calcium detection together with lumen and external elastic lamina measurements, improving the efficiency and reproducibility of image interpretation. In a multireader study involving 30 operators, AI-assisted analysis improved the accuracy of calcium severity assessment and vessel preparation strategy selection while reducing interobserver variability and analysis time [77].

At present, AI-assisted analysis should be regarded as a decision-support tool rather than a replacement for expert interpretation. Although these technologies improve workflow and standardize image analysis, evidence demonstrating an impact on procedural or long-term clinical outcomes remains limited. As hardware and software continue to evolve, HF-OCT and AI-assisted analysis have the potential to further facilitate the adoption of OCT-guided PCI in complex calcified coronary disease.

## 9. Multimodality Comparison and Imaging-Guided Device Selection

No single imaging modality provides a complete assessment of calcified coronary lesions. Each technique contributes distinct information across different stages of evaluation: coronary angiography provides initial anatomical assessment and procedural guidance, CCTA enables noninvasive whole-coronary assessment and preprocedural planning, IVUS provides robust evaluation of vessel architecture and calcium distribution, NIRS-IVUS adds information regarding plaque composition, and OCT/HF-OCT offers the highest spatial resolution for detailed calcium morphology and thickness assessment. The complementary strengths and limitations of these modalities are summarized in Table 1, while validated intracoronary calcium scores associated with stent underexpansion are presented in Table 2.

**Figure 1:**
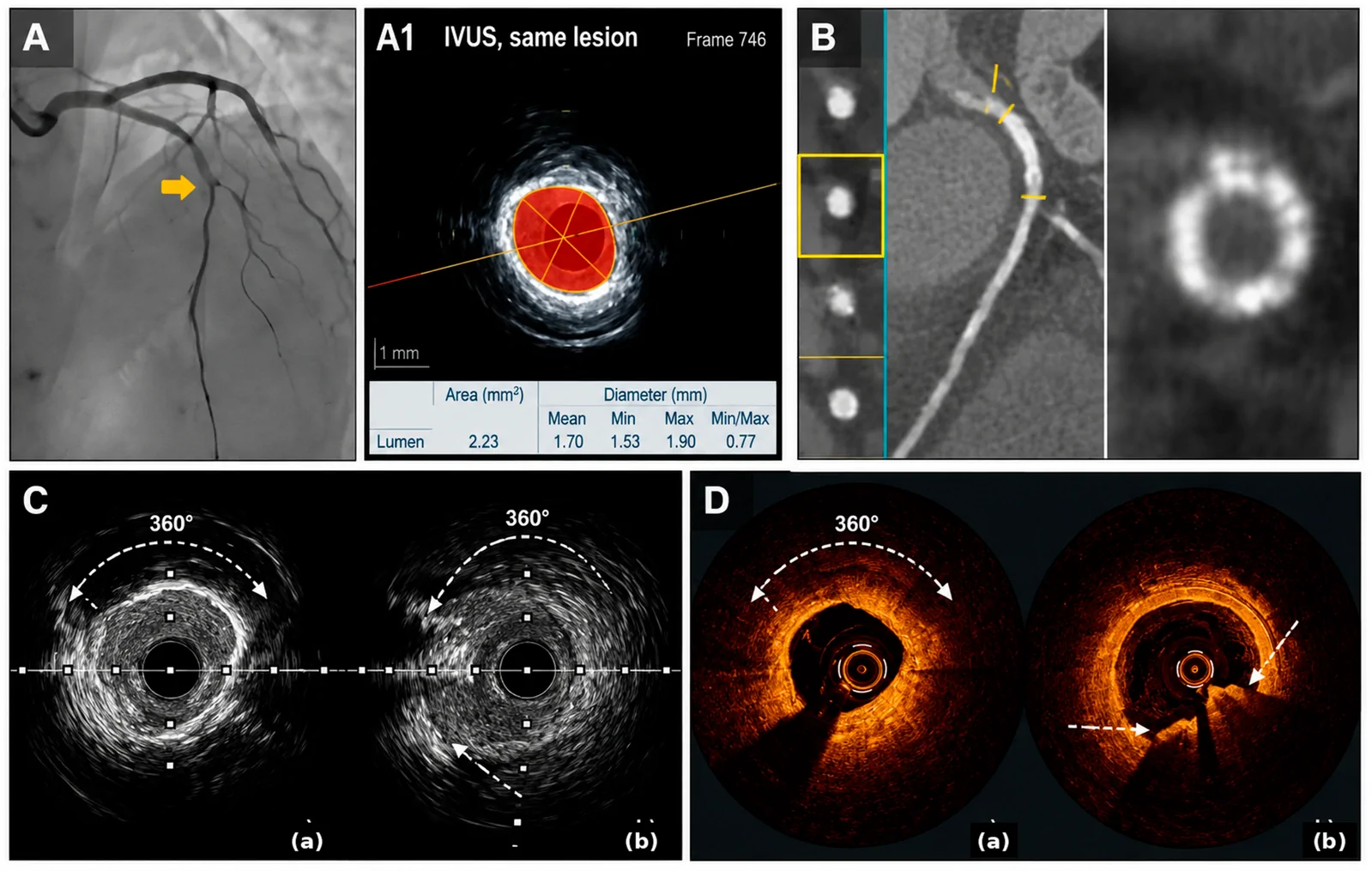
Appearance of coronary calcium on different imaging modalities. (**A**) Invasive coronary angiography of a mid-left anterior descending artery stenosis (arrow) in which no calcium is discernible. (**A1**) Intravascular ultrasound (IVUS) of the same lesion demonstrates 360° circumferential calcification despite the absence of angiographically visible calcium, illustrating the limited sensitivity of angiography for coronary calcium. On angiography, calcium is visible only as linear radiopacities along the vessel wall before contrast injection and only when sufficiently dense; its arc, thickness, and depth cannot be measured. (**B**) Coronary CT angiography: curved multiplanar reconstruction and cross-section of a heavily calcified proximal–mid-left anterior descending artery showing semicircumferential to near-concentric calcium; blooming artifact exaggerates calcium dimensions and obscures the residual lumen. The yellow box identifies the selected cross-sectional level, while the yellow reference markers indicate the corresponding lesion segment on the curved multiplanar reconstruction. (**C-a**) Intravascular ultrasound showing 360° circumferential calcium as a bright hyperechoic arc with posterior acoustic shadowing, which precludes measurement of calcium thickness and assessment of tissue behind the leading edge. (**C-b**) Intravascular ultrasound showing an eruptive calcified nodule protruding into the lumen. (**D-a**) Optical coherence tomography showing 360° circumferential calcium as a signal-poor, heterogeneous region with sharply delineated borders and no posterior shadowing, allowing direct measurement of calcium arc, thickness, and length. The dashed curved arrow indicates the circumferential calcium arc. (**D-b**) Optical coherence tomography showing a calcified nodule with an irregular surface protruding into the lumen; the dashed arrows indicate the protruding calcified nodule. All panels are reproduced from open-access publications under the Creative Commons Attribution (CC BY) license: (**A**) from Cui et al. [78]; (**B**) from Fargione et al. [64]; and (**C**,**D**) from Kałmucki et al. [79].

The intravascular modalities also differ in field of view and penetration, which determine what each can measure. IVUS images the entire vessel wall to the adventitia and beyond (penetration 5–10 mm; field of view approximately 10–15 mm in diameter), which is why it is suitable for large vessels and the left main artery and can assess vessel remodelling even in the presence of extensive plaque. NIRS is acquired on the same catheter and shares the IVUS field of view for co-registration but interrogates only the innermost approximately 1 mm of the wall, sufficient to assess lipid core beneath the lumen but unable to assess deeper tissue. The field of view of OCT (approximately 10 mm in diameter) is adequate for most coronary segments, but its penetration of only 1–2.5 mm, reduced further by highly attenuating tissue such as lipid, means that the outer border of deep or thick calcium and the external elastic lamina are frequently not visualized. Calcium thickness, therefore, cannot always be measured in exactly those lesions in which it matters most, and vessel sizing is less reliable in large or ectatic vessels. HF-OCT retains the resolution of OCT with a scan range accommodating vessels from 1.3 to 6.0 mm in diameter [75]. The appearance of coronary calcium on each modality is illustrated in Figure 1.

**Figure 2:**
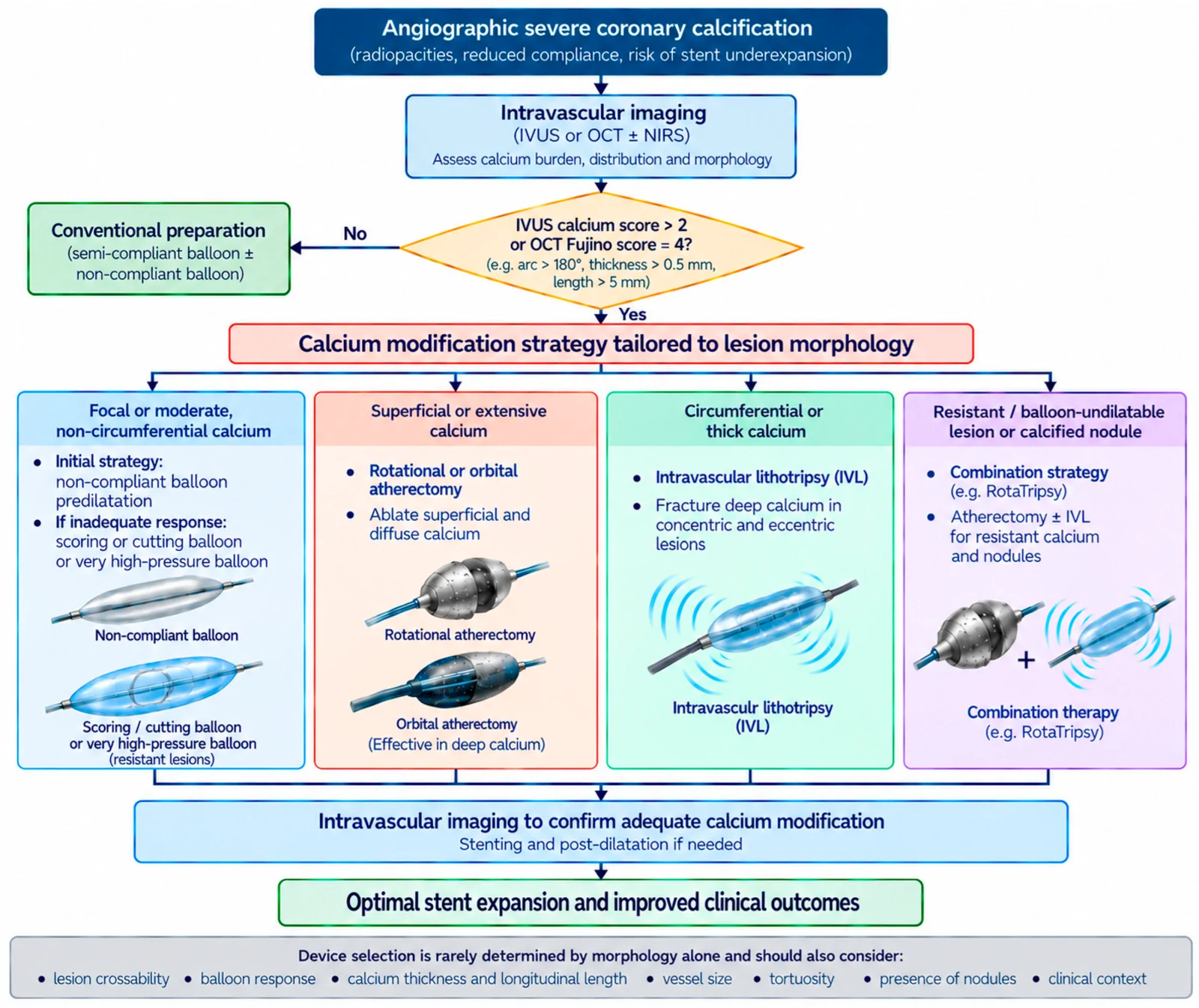
Proposed imaging-guided framework for selecting a calcium-modification strategy, synthesized from contemporary consensus documents and available evidence. Severe angiographic calcification prompts intracoronary imaging. IVUS- and OCT-derived calcium scores identify lesions at increased risk of stent underexpansion. Calcium morphology then informs device selection: atherectomy may be considered for superficial or extensive calcification, intravascular lithotripsy for circumferential or thick calcium, conventional non-compliant balloon predilatation for focal, non-circumferential calcium (with scoring, cutting, or super-high-pressure balloons considered if the response is inadequate), and combination approaches for resistant, balloon-undilatable lesions. Device selection depends additionally on lesion crossability, the response to initial balloon dilatation, calcium thickness and length, vessel size, tortuosity, and the presence of a calcified nodule (Box). This framework is consensus-based and has not been prospectively validated against clinical outcomes; it is intended as a conceptual aid rather than a prescriptive algorithm. IVUS, intravascular ultrasound; OCT, optical coherence tomography.

Although these scores reliably identify lesions at increased risk of stent underexpansion, they were developed primarily as risk stratification tools and prospective studies are needed to determine whether score-guided device selection improves clinical outcomes. These imaging parameters provide the basis for a morphology-guided approach to calcium modification (Figure 2). According to the 2024 SCAI Expert Consensus, severe angiographic calcification should prompt further evaluation with intracoronary imaging, while high-risk calcium features identified by IVUS or OCT may indicate lesions that could benefit from upfront calcium modification [44,80]. Importantly, these imaging criteria are currently supported by mechanistic and observational evidence, but prospective trials directly demonstrating improved clinical outcomes with imaging-guided device selection remain limited.

Applied clinically, imaging findings can assist in matching lesion morphology with the most appropriate calcium-modification strategy. Superficial, concentric, or extensive calcification may favor rotational or orbital atherectomy, which primarily modifies superficial intimal calcium through mechanical plaque ablation [81,82,83,84,85]. Deep or circumferential calcium may be more suitable for intravascular lithotripsy, which generates acoustic pressure waves capable of producing fractures within both superficial and deep calcium deposits [86,87,88,89]. Focal, moderate, and non-circumferential calcification is generally approached first with conventional non-compliant balloon predilatation, which remains a standard initial approach; scoring or cutting balloons, or super-high-pressure balloons, may be considered when the response to conventional balloon dilatation is inadequate [90,91]. Finally, lesions that remain balloon-undilatable or demonstrate persistent deep calcium despite initial modification may require combination strategies, including RotaTripsy [92,93].

Device selection in practice is rarely determined by calcium morphology alone. Lesion crossability governs whether an atherectomy burr, lithotripsy balloon or even an imaging catheter can be delivered, and uncrossable lesions may require rotational atherectomy or a stepwise small-balloon approach before any imaging is possible. The response to initial balloon dilatation (full expansion, residual waist or balloon rupture) is itself diagnostic and frequently redirects the strategy. Calcium thickness (with thicker calcium being more resistant to balloon-based fracture), calcium length (long segments may favor atherectomy), vessel size (large vessels may limit the efficacy of small burrs and favor lithotripsy; small vessels may limit lithotripsy balloon availability), tortuosity and angulation (which limit the deliverability of lithotripsy balloons and may increase procedural complexity and the risk of complications with atherectomy), and the presence of a calcified nodule (for which atherectomy may be particularly useful, although the optimal modification strategy remains uncertain) all modify the choice. The framework in Figure 2 is therefore a conceptual aid synthesized from consensus documents [44,94], not a validated algorithm; no randomized trial has tested morphology-guided device selection against operator judgment, and the framework should be applied with these additional determinants in mind.

Current guidelines and consensus documents increasingly support this imaging-informed and morphology-guided approach to calcified lesion preparation. The 2021 ACC/AHA/SCAI guideline assigns rotational atherectomy a Class IIa recommendation, while other calcium-modification devices receive Class IIb recommendations [95]. More recent SCAI, EAPCI, and ESC consensus documents emphasize the importance of intracoronary imaging for calcium characterization before and after lesion modification, although the optimal device-selection strategy remains dependent on lesion morphology, operator expertise, and available technology [44,94,96].

## 10. Impact of Imaging-Guided PCI on Outcomes

The clinical rationale for detailed coronary calcium assessment is that imaging-guided PCI allows more accurate procedural optimization and correction of angiographically occult limitations. The principal mechanism through which coronary calcification adversely affects PCI is inadequate lesion preparation and subsequent stent underexpansion, which are strongly associated with stent thrombosis, restenosis, and adverse clinical outcomes [2]. An OCT-derived stent expansion index below 0.86 has independently been associated with major adverse cardiovascular events [74]. By enabling accurate assessment of vessel dimensions, lesion preparation, stent deployment, and residual mechanical complications, intravascular imaging provides information that cannot be reliably obtained from angiography alone.

In interpreting this evidence, it is important to distinguish randomized trials of intravascular imaging guidance per se, which are now numerous, from observational data and subgroup analyses concerning calcified lesions specifically, which remain the only evidence for several of the morphology-guided strategies discussed previously. A substantial body of randomized evidence supports the use of IVUS-guided PCI. The IVUS-XPL trial demonstrated a sustained reduction in major adverse cardiac events at five years among patients undergoing PCI for long coronary lesions compared with angiography-guided intervention [66]. The ULTIMATE trial further demonstrated a reduction in target vessel failure with IVUS-guided drug-eluting stent implantation in an all-comers population, with the greatest benefit observed when predefined criteria for an optimal IVUS-guided result were achieved [97]. A network meta-analysis of randomized trials confirmed that intravascular imaging-guided drug-eluting stent implantation is associated with reductions in target lesion failure, cardiac death, and stent thrombosis compared with angiography-guided PCI [67].

The major randomized trials and meta-analyses evaluating the clinical impact of intravascular imaging-guided PCI are summarized in Table 3.

Evidence supporting OCT-guided PCI has also expanded. The ILUMIEN IV trial, which enrolled high-risk patients and complex lesions, showed that OCT guidance produced a larger minimal stent area (5.72 vs. 5.36 mm^2^, *p* < 0.001), but this did not translate into a statistically significant reduction in the primary clinical endpoint of target vessel failure at two years (7.4% vs. 8.2%, *p* = 0.45); definite or probable stent thrombosis, a secondary endpoint, was lower with OCT guidance (0.5% vs. 1.4%) [98]. The OCTOBER trial demonstrated improved two-year clinical outcomes with OCT guidance in complex bifurcation lesions, highlighting the value of detailed anatomical assessment in challenging interventions [99]. The OCTIVUS trial subsequently demonstrated that OCT-guided PCI was non-inferior to IVUS-guided PCI for one-year clinical outcomes, suggesting that both imaging modalities can provide effective procedural guidance when appropriately applied [100].

The relevance of intravascular imaging is particularly evident in the complex and calcified lesions that represent the focus of this review. The RENOVATE-COMPLEX-PCI trial, in which heavily calcified lesions constituted a pre-specified lesion subset, demonstrated improved outcomes with intravascular imaging-guided PCI in complex coronary interventions [101]. The ECLIPSE trial, by contrast, addressed lesion preparation rather than imaging guidance: 2005 patients with severely calcified lesions were randomized to orbital atherectomy versus balloon angioplasty before DES implantation, and orbital atherectomy neither increased the minimal stent area (7.67 vs. 7.42 mm^2^, *p* = 0.078) nor reduced one-year target vessel failure (11.5% vs. 10.0%, *p* = 0.28) [102]. Intravascular imaging was recommended but not mandated in ECLIPSE. In a post hoc, non-randomized analysis, the 62% of patients treated with imaging guidance had a lower adjusted rate of target vessel failure than those treated with angiographic guidance alone (9.3% vs. 13.2%; adjusted hazard ratio 0.74, 95% CI 0.56–0.97) [103]. Because imaging was used at the operator’s discretion, this association is subject to confounding by indication and should be regarded as hypothesis-generating support, consistent with the randomized trials of imaging guidance in complex PCI, rather than as randomized proof of benefit in calcified lesions. Dedicated randomized trials of imaging-guided calcium modification are still lacking, and intravascular imaging should therefore be regarded as an active component of PCI optimization whose benefit in calcified lesions is inferred from randomized trials in broader complex-PCI populations and from observational data in calcified lesions specifically.

Despite increasing evidence, real-world adoption of intravascular imaging remains limited. In a large United States analysis of single-vessel CTO PCI, IVUS use increased from 6% of procedures in 2008 to 18% in 2020, with OCT use remaining below 10% [104]. This persistent gap between evidence and clinical implementation highlights the need for continued efforts to integrate intravascular imaging into routine practice, particularly for patients with complex and heavily calcified coronary disease.

## 11. Barriers to Implementation

The barriers to imaging adoption are structural rather than evidentiary. Economic disincentives, workflow constraints, uneven distribution of expertise, ambiguous selection criteria, and interpretive variability each operate independently, and addressing any one in isolation is unlikely to close the implementation gap. Progress will require coordinated action across reimbursement policy, training structures, technology development, and guideline specificity, alongside the prospective trials needed to validate imaging-guided treatment algorithms.

## 12. Future Directions

Future advances in the imaging of calcified coronary lesions are likely to arise from continued improvements in both imaging technology and image interpretation. Rather than replacing existing imaging modalities, these developments aim to improve the efficiency, reproducibility, and accessibility of imaging-guided PCI.

AI-assisted image analysis is being developed to automate calcium detection, segmentation, and quantification across OCT, IVUS, and CCTA. These technologies have the potential to standardize image interpretation, reduce interobserver variability, and improve workflow efficiency, although current evidence remains largely limited to feasibility and validation studies [105,106,107,108,109]. Whether AI-assisted analysis translates into improved procedural decision-making or better clinical outcomes remains to be established.

At the same time, ongoing hardware innovations continue to expand the capabilities of coronary imaging. High-frequency OCT seeks to improve catheter deliverability and image acquisition in complex coronary anatomy. In parallel, CT-guided PCI planning is moving from feasibility to randomized evaluation; the P4 trial will evaluate whether CCTA-based planning can be non-inferior to IVUS guidance, and its results may shape how calcium assessment is distributed between the preprocedural and intra-procedural stages.

Future research should focus on prospective studies evaluating whether imaging-derived calcium scores, morphology-guided device selection, and AI-assisted image interpretation improve procedural efficiency and long-term clinical outcomes. Establishing standardized imaging protocols and validating imaging-guided treatment algorithms will be essential before these technologies can be routinely incorporated into clinical practice.

## 13. Limitations

This review has several limitations. First, it is a narrative rather than a systematic review; consequently, although the literature search was comprehensive, the absence of a predefined protocol, formal study selection process, and quantitative quality assessment introduces the potential for selection bias. Second, the available evidence is heterogeneous, comprising randomized trials, observational studies, registry data, expert consensus documents, and imaging validation studies with differing methodologies and clinical endpoints.

In addition, the evidence supporting newer imaging technologies, including NIRS-IVUS in calcified coronary disease, high-frequency OCT, and AI-assisted image analysis, remains limited and is derived predominantly from feasibility studies, observational cohorts, or early clinical experience rather than large randomized outcome trials.

Finally, although contemporary consensus documents increasingly advocate morphology-guided calcium modification based on intracoronary imaging, many proposed imaging algorithms and device-selection strategies have not yet been prospectively validated in dedicated randomized studies. Further prospective investigations are needed to determine whether imaging-guided treatment algorithms translate into improved long-term clinical outcomes.

## 14. Conclusions

Population aging is sharply increasing the prevalence of severely calcified lesions during PCI. The assessment of calcified coronary lesions has evolved from a predominantly angiographic evaluation to a comprehensive multimodality imaging approach that enables detailed characterization of calcium burden, morphology, and plaque composition. Rather than competing, contemporary imaging modalities provide complementary information that supports lesion assessment before PCI, optimizes procedural planning, and facilitates the evaluation of the final procedural result; for intravascular imaging guidance, randomized trials in complex PCI have shown that this translates into improved clinical outcomes.

Among invasive imaging techniques, IVUS and OCT have become central to contemporary calcium assessment, with validated imaging-derived calcium scores allowing the identification of lesions at increased risk of stent underexpansion and supporting a more individualized approach to lesion preparation. Emerging technologies, including high-frequency OCT and AI-assisted image analysis, may further improve the efficiency and reproducibility of intracoronary imaging, although prospective clinical validation remains necessary.

Noninvasive CT-guided PCI planning is the most significant near-term development. At present, CCTA-based PCI planning has been endorsed by SCAI and SCCT, and randomized evidence of procedural benefit exists for CTO PCI; however, CT-based plans still require intravascular confirmation once the lesion has been prepared. In the near future, the P4 trial will evaluate whether CT-guided PCI is non-inferior to IVUS-guided PCI with respect to clinical outcomes [76]. If non-inferiority is demonstrated, and as photon-counting CT and AI-based plaque analysis mature, much of the calcium assessment described in this review may shift to the preprocedural stage, with intravascular imaging retained to confirm calcium modification and to optimize the final stent result.

Current evidence supports a shift from angiography-guided intervention toward imaging-informed, morphology-guided PCI. Randomized trials have established that intravascular imaging guidance improves outcomes in complex PCI, with calcified lesions represented within these populations; whether morphology-guided selection of calcium-modification devices confers additional benefit remains to be demonstrated in dedicated randomized trials. Continued integration of multimodality imaging into routine clinical practice, together with the emerging role of CT-guided planning, has the potential to guide calcium-modification strategies, improve procedural outcomes, and ultimately enhance the care of patients with complex calcified coronary artery disease.

## Figures and Tables

**Table 1:** Comparison of imaging modalities for coronary calcium assessment.

Modality	Resolution	Calcium Assessment	Principal Strengths	Principal Limitations
Coronary angiography	~0.2 mm (2D projection)	Radiopacity; detects ~40–50% of calcium identified by IVUS/OCT [1,8]	Universal, real time	Underestimates arc and depth; interobserver variability
CCTA	0.4–0.6 mm (energy-integrating CT); 0.11–0.2 mm (photon-counting CT, ultra-high-resolution mode)	Agatston score; 3D volume (overestimates ~60% vs. OCT)	Noninvasive, whole tree, preprocedural planning	Blooming artifact; limited lumen detail in severe calcium
IVUS	Axial 100–150 μm (40 MHz)/40–60 μm (60 MHz); lateral 150–300 μm; penetration 5–10 mm; field of view ~10–15 mm	Arc, longitudinal length and distribution; thickness not measurable; deep calcium behind superficial calcium not assessable	Deep penetration; robust; Zhang score	Acoustic shadowing: cannot measure calcium thickness or assess deep calcium and tissue behind the leading edge of superficial calcium
NIRS-IVUS	As IVUS; NIRS penetration ~1 mm (chemogram, no structural resolution)	IVUS metrics plus lipid core (maxLCBI); nodule composition	Adds lipid characterization; flags high-risk nodules	Same shadowing as IVUS (no thickness or deep calcium behind superficial calcium); NIRS interrogates only ~1 mm depth
OCT or HF-OCT	Axial 10–20 µm; lateral 20–40 µm; penetration 1–2.5 mm; field of view ~10 mm. HF-OCT: axial ≤20 µm; scan range 1.3–6.0 mm vessels	Arc, length, and thickness; Fujino and Sato scores	Highest resolution; direct thickness; HF-OCT deliverability	Limited penetration in thick calcium; needs blood clearance

**Table 2:** Validated intravascular imaging calcium scores predicting stent underexpansion.

Score	Modality	Components	High-Risk Threshold	Predicts
Zhang	IVUS	Superficial calcium arc >270° extending over >5 mm (1 pt); 360° superficial calcium (1 pt); calcified nodule (1 pt); reference vessel diameter <3.5 mm (1 pt); range 0–4	Score ≥ 2	Stent underexpansion (stent expansion < 70%)
Fujino	OCT	Maximum calcium angle >180° (2 pts); maximum calcium thickness >0.5 mm (1 pt); calcium length >5 mm (1 pt); range 0–4	Score of 4 (stent expansion 78% vs. 96% for scores 0–3)	Stent underexpansion
Sato revised	OCT	Calcium arc >270° with length >3 mm (1 pt); 360° calcium (1 pt); minimum calcium thickness >0.30 mm (1 pt); range 0–3	Score 3; stent expansion decreases stepwise with each additional point	Stent underexpansion (stent expansion < 70%) in severely calcified lesions in the contemporary DES era

**Table 3:** Major clinical trials evaluating the impact of intravascular imaging-guided PCI.

Study	Imaging Modality	Population	Main Finding	Clinical Implication
IVUS-XPL (RCT)	IVUS	PCI for long coronary lesions	IVUS-guided PCI reduced major adverse cardiac events with sustained benefit at long-term follow-up	Supports IVUS-guided optimization in complex coronary lesions
ULTIMATE (RCT)	IVUS	All-comers undergoing drug-eluting stent implantation	IVUS-guided PCI reduced target vessel failure compared with angiography guidance	Supports routine IVUS optimization when feasible
Network meta-analysis of RCTs	IVUS/OCT	Randomized trials of intravascular imaging-guided PCI	Imaging-guided PCI reduced target lesion failure, cardiac death, and stent thrombosis compared with angiography guidance	Confirms overall clinical benefit of intravascular imaging
ILUMIEN IV (RCT)	OCT	High-risk patients and complex lesions	OCT guidance increased minimal stent area (5.72 vs. 5.36 mm^2^) but did not significantly reduce 2-year target vessel failure (7.4% vs. 8.2%, *p* = 0.45); definite/probable stent thrombosis lower (0.5% vs. 1.4%, secondary endpoint)	A better procedural result did not translate into the primary clinical endpoint; supports OCT for stent optimization, with clinical benefit unproven in this trial
OCTOBER (RCT)	OCT	Complex bifurcation lesions	OCT-guided PCI improved two-year clinical outcomes compared with angiography guidance	Supports OCT use in anatomically complex lesions
OCTIVUS (RCT)	OCT vs. IVUS	Patients undergoing PCI	OCT-guided PCI was non-inferior to IVUS-guided PCI for one-year clinical outcomes	Suggests both modalities provide effective procedural guidance
RENOVATE-COMPLEX-PCI (RCT)	IVUS/OCT	Complex coronary lesions	Intravascular imaging-guided PCI reduced adverse cardiovascular events compared with angiography guidance	Supports imaging use in complex PCI
ECLIPSE (RCT of lesion preparation) and ECLIPSE imaging analysis (non-randomized)	IVUS or OCT at operator discretion (62.1% of patients)	Severely calcified lesions randomized to orbital atherectomy vs. balloon angioplasty	Randomized comparison: orbital atherectomy did not increase minimal stent area (7.67 vs. 7.42 mm^2^) or reduce 1-year target vessel failure (11.5% vs. 10.0%). Non-randomized comparison: imaging-guided PCI was associated with lower adjusted target vessel failure than angiography-guided PCI (9.3% vs 13.2%, adjusted HR 0.74)	Observational support for imaging guidance in calcified lesions; no randomized evidence of imaging benefit specific to calcified lesions

## Data Availability

No new data were created or analyzed in this study. Data sharing is not applicable to this article.

## Abbreviations

The following abbreviations are used in this manuscript:

18F-NaF PET	18F-sodium fluoride positron emission tomography
2D	Two-dimensional
3D	Three-dimensional
ACC	American College of Cardiology
AHA	American Heart Association
AI	Artificial intelligence
CAC	Coronary artery calcium
CCTA	Coronary computed tomography angiography
CT	Computed tomography
CT-FFR	Computed-tomography-derived fractional flow reserve
CTO	Chronic total occlusion
DES	Drug-eluting stent
EAPCI	European Association of Percutaneous Cardiovascular Interventions
ESC	European Society of Cardiology
HF-OCT	High-frequency optical coherence tomography
HU	Hounsfield units
IVUS	Intravascular ultrasound
LCBI	Lipid core burden index
maxLCBI4mm	Maximum lipid core burden index over 4 mm
MESA	Multi-Ethnic Study of Atherosclerosis
MHz	Megahertz
NIRS	Near-infrared spectroscopy
NIRS-IVUS	Near-infrared spectroscopy intravascular ultrasound
OCT	Optical coherence tomography
PCCT	Photon-counting detector computed tomography
PCI	Percutaneous coronary intervention
SCAI	Society for Cardiovascular Angiography and Interventions
SCCT	Society of Cardiovascular Computed Tomography
